# The rising use of cognitive enhancement drugs and predictors of use during COVID-19: findings from a cross-sectional survey of students and university staff in the UK

**DOI:** 10.3389/fpsyg.2024.1356496

**Published:** 2024-07-15

**Authors:** Jamie L. Tully, Oliver Bridge, Joseph Rennie, Joy Krecké, Tobias Stevens

**Affiliations:** ^1^Department of Psychology, University of Exeter, Exeter, United Kingdom; ^2^School of Education, University of Exeter, Exeter, United Kingdom

**Keywords:** cognitive enhancement, psychoactive drugs, predictors of drug use, personality factors, microdosing, psychedelics, COVID-19

## Abstract

**Background:**

The use of psychoactive substances to increase cognitive performance while studying has been termed ‘pharmacological cognitive enhancement’ (PCE). In previous years, several large-scale national surveys have focused on their use by students at university, including drug types, prevalence rates, and predictive factors. The recent coronavirus pandemic brought about widespread structural changes for UK universities, as students were forced to adapt to home-based learning and in many cases reduced academic support. No study has yet focused primarily on the impact of pandemic social restrictions on PCE in students and academic staff, and whether personality and demographic factors reveal user profiles that predict use during the pandemic period.

**Method:**

A convenience sample of 736 UK students and staff aged 18–54 (*M* = 22.2, *SD* = 5.2) completed a cross-sectional survey assessing PCE prevalence rates, polydrug use, perceived effects, academic self-efficacy and personality during the first year of social restrictions (March 2020 – February 2021) compared with the previous year (March 2019 – February 2020).

**Results:**

There was a significant self-reported rise in the use of all drug types (all *p*s < 0.001) during social restrictions, particularly with Modafinil (+42%), nutraceuticals (+30.2%) and microdose LSD (+22.2%). Respondents also indicated stronger PCE effects for all substances, except alcohol, in comparison to the previous year. Polydrug use with modafinil and other prescription stimulants increased the most during social restrictions. Personality factors and gender identity reliably predicted PCE use and lower agreeableness was often the strongest predictor, followed by identifying as male and lower conscientiousness. Academic self-efficacy and student/academic staff status were not consistent predictors.

**Conclusion:**

This is the first survey of UK students to investigate PCE during coronavirus social restrictions and to assess predictive factors. Findings reveal a rise in PCE use and polydrug use which we suggest is because of increased pressures on students created by the lockdown and reduced access to university resources.

## 1 Introduction

The use of illicit and prescription drugs for the purposes of pharmacological cognitive enhancement (PCE) is increasing, particularly in the higher education context globally (Banjo et al., 2010; Sattler et al., 2013; Singh et al., 2014; Liakoni et al., 2015; Maier et al., 2015a, 2018; Lengvenyte et al., 2016; Mousavi et al., 2019; Monnet et al., 2021; Ram et al., 2021). Maier et al. (2018) define pharmacological neuroenhancement as “the [non-medical] use of prescription drugs, alcohol, and illegal drugs for the purposes of enhancing cognition, mood, or pro-social behaviour to improve performance at work or while studying [by healthy individuals]” (p.1). They further qualify pharmacological cognitive enhancement (PCE) as the non-medical use of drugs by people who are healthy or (at least) with an undiagnosed condition with the intention to augment their cognitive performance for work or studying.

Such drugs can be further divided into easily available ‘soft-enhancers’ (e.g., caffeine, vitamins, energy drinks, and nutraceuticals, which are popular herbal products promoted for their health benefits and can be purchased as over-the-counter medicines), and ‘hard-enhancers’ such as the non-medical use of prescription drugs (e.g., modafinil, methylphenidate, dextroamphetamine) by healthy individuals and illicit drugs (e.g., cocaine, LSD, psilocybin) (Liakoni et al., 2015). These substances are used in order to improve concentration (Mousavi et al., 2019) and focus (Stewart et al., 2019), creativity (Prochazkova et al., 2018; Anderson et al., 2019), memory (Repantis et al., 2010; Brunner et al., 2017), alertness (Teter et al., 2005; Bossaer et al., 2013), mood (Kraehenmann et al., 2015), motivation (D’Angelo et al., 2017), and/or overall cognitive performance (Sharif et al., 2021). Hard PCE usage has been increasing over the previous decades, and this is increasingly visible in higher education settings in the West (Banjo et al., 2010; Sattler et al., 2013; Singh et al., 2014; Liakoni et al., 2015; Maier et al., 2015a; Lengvenyte et al., 2016; Maier et al., 2018; Monnet et al., 2021; Ram et al., 2021).

In this paper we focus on the use of PCE strategies within a higher education context and explore their use during the beginning period of the recent coronavirus pandemic. It is crucial to keep track of the use of PCEs in higher education, as there are several ethical issues associated with hard PCE usage, including concerns around the short and long term safety of PCE and the issues related to fairness. Concerning the potential long term impacts of sustained PCE usage, there is relatively little research (Steiner and van Waes, 2013). Sahakian and Morein-Zamir (2010) argue that neuropsychiatric patients may tolerate the adverse side effects of PCEs since the benefit they get from using these drugs outweigh the negative side effects, but that the same justification does not apply to healthy individuals, and call for more research into this area.

More importantly, the lockdown measures to combat the spread of the pandemic have been associated with an increase in depressive symptoms and higher consumption of psychoactive substances (Dogan-Sander et al., 2021; Jodczyk et al., 2022). The same measures have also led to reduced academic support in HE settings, and poor academic performance has been linked to PCE usage (Verdi et al., 2016). This is of particular importance, because there has been an observed increase in the harmful side effects of substance use during the lockdowns, including fatal side effects, due to increased toxicity stemming from a lack of regulation (Macmadu et al., 2021; Nguyen and Buxton, 2021; Friedman and Hansen, 2022). The combination of an increase in mental health issues, reduced academic support, and access to PCEs through unregulated/less well-regulated sources raises concerns regarding adverse side effects on regular PCE users.

Finally, PCE usage also raises fairness related concerns. PCE use is more prevalent in highly competitive academic environments (see below), and to the degree that access to PCEs is mediated by socioeconomic factors, this has the potential to widen the gap between individuals who possess greater advantages and those who do not. This same concern also applies more broadly in international contexts as well (Sahakian and Morein-Zamir, 2010), potentially widening international inequalities. Besides equality concerns stemming from socioeconomic mediators for access, regular use of PCEs have also raised concerns regarding authenticity of performance (Bostrom and Sandberg, 2009), an over-reliance on PCEs eroding virtues such as hard working and motivation (Sahakian and Morein-Zamir, 2010), and “academic doping” (Maher, 2008; Garasic and Lavazza, 2016).

Estimates of PCE usage prevalence among university students vary widely between studies depending on methodology, sample size and characteristics, such as whether the sample contains only students or members of the general population as well, whether the sample consists of students of specific disciplines (e.g., medicine), and whether soft enhancers such as caffeine are considered in the study. Some of the highest and lowest estimates among UK university students vary from just 0.5% (Holloway and Bennett, 2011) to 48% when caffeine is included (Hanna et al., 2018). In studies conducted elsewhere in Europe, estimates have varied from just 4.7% among Swiss students (Ott and Biller-Andorno, 2013) to 74.7% among Italian medical students (Pighi et al., 2018). In North America, similar estimates include only 2% usage in Canadian medical students (Kudlow et al., 2013), to 75.8% of American students in a particular institution (Teter et al., 2006). Similar studies across the rest of the world have found around 6% prevalence among Australian university students (Riddell et al., 2017; Lucke et al., 2018), between 2.9% (Abbasi Ghahramanloo et al., 2015) and 17.6% (Mousavi et al., 2019) in Iran, and between 4.2% (de Oliveira Cata Preta et al., 2020) and 5.8% (Cândido et al., 2020) among students in Brazil (See Sharif et al., 2021 for a comprehensive overview). Maier et al. (2018) document that the use of prescription stimulants increased from 3.2% in the Global Drug Survey 2015 report to 6.6% in the 2017 report. Overall, there appears to be a clear upward trend in recent decades, particularly among highly competitive courses and higher education institutions.

An associated phenomenon is the use of multiple substances simultaneously (overlapping) or concurrently (not overlapping), termed polydrug use, or stacking, for recreational or enhancement purposes. This can include using soft enhancers, prescription stimulants and/or illicit drugs together. While the adverse effects of using cognitive enhancers are rare, combinations of different substances increase such risks (Tully et al., 2019). Research into stacking is more often in the context of substance use disorders (e.g., Salom et al., 2015; John et al., 2018; Hiebler-Ragger and Unterrainer, 2019), or in the context of use among adolescents (e.g., Rodríguez-Arias and Aguilar, 2012; Patrick et al., 2017; Bello et al., 2019; Zuckermann et al., 2019) rather than cognitive enhancement. Non-medical use of prescription drugs has been associated with a greater prevalence of stacking (Schelle et al., 2015; Riddell et al., 2017; Ponnet et al., 2021). Zuckermann et al. (2019) found a 50% increase in poly-substance use in Canadian secondary school students during the 2013–2018 period, with a sharp rise after 2016, and Patrick et al. (2017) report up to 21% of 12th graders in the US between 1976 and 2016 used alcohol and cannabis simultaneously, evidencing a cultural history of stacking. Bello et al. (2019) found that adolescents from lower socioeconomic backgrounds were at greater risk of stacking. In the context of higher education, Schelle et al. (2015) found that students at Dutch universities that used prescription drugs for CE purposes were more likely to engage in stacking than those who used prescription drugs for non-CE purposes, and Riddell et al. (2017) report that Australian students who use any set of substances for cognitive enhancement are more likely to stack drugs for CE purposes, however neither paper goes into detail about which specific substances are stacked, or how.

There is also a growing literature on how personality and values relate to PCE usage (Kotov et al., 2010; Benotsch et al., 2013; Sattler and Schunck, 2016; Maier et al., 2018; Mayor et al., 2020; Dash et al., 2021; Grinschgl et al., 2022; Schönthaler et al., 2022). Several studies have been conducted to measure the relationship between PCE or more generally substance mis/use (Kotov et al., 2010; Maier et al., 2018) and the Five Factor Model of personality (Turiano et al., 2012; Benotsch et al., 2013; Sattler and Schunck, 2016; Dash et al., 2021; Schönthaler et al., 2022), as well as other studies investigating other personality traits (Maier et al., 2015b, 2018; Mayor et al., 2020; Grinschgl et al., 2022; Schönthaler et al., 2022). The Five Factor Model is one of the most robust measurements of personality currently available, and covers five main traits: extraversion, conscientiousness, neuroticism, openness to new experiences, and agreeableness (McCrae and Costa, 1987).

The picture that appears to be emerging from these studies point to a particular personality profile as at greater risk of mis/using PCE (Maier et al., 2018). Conscientiousness and neuroticism have one of the most robust relationships with drug and cognitive enhancer use and are widely reported. Lower levels of conscientiousness predict prior drug and cognitive enhancer use (Kotov et al., 2010; Sattler and Schunck, 2016; Dash et al., 2021), whereas higher levels of neuroticism have been linked to higher use of PCEs (Kotov et al., 2010; Benotsch et al., 2013; Sattler and Schunck, 2016). The relationship between agreeableness and PCE use is less robust, however the general picture indicates that lower levels of agreeableness predict an increase in drug use (Kotov et al., 2010; Turiano et al., 2012), although the findings are less robust specifically regarding cognitive enhancer use, as Sattler and Schunck (2016) did not find any relationship between agreeableness and PCE use, and Schönthaler et al. (2022) found that high agreeableness is negatively related to acceptance of self-enhancement overall. Findings on openness are somewhat more mixed, as reports indicate that openness predicts drug use in general (Turiano et al., 2012), and PCE use among younger people specifically (Benotsch et al., 2013), but also predicts negative attitudes toward enhancement in general (Grinschgl et al., 2022). Finally, the relationship between extraversion and PCE and drug use are less clear, as Sattler and Schunck (2016) report that extraversion had no relationship with cognitive enhancer use while Turiano et al. (2012) found that extraversion predicts longitudinal use of drugs more generally, and Dash et al. (2021) report that extraversion is specifically related to cocaine/crack and stimulant use.

Despite the fact that PCE is used often among some higher education students, relatively little research has been done linking academic self-efficacy and PCE usage. Overall, extant research indicates that students with lower marks are more likely to use PCE and/or have a greater willingness to use PCE in order to improve their academic performance (McCabe et al., 2005; Rabiner et al., 2008; Franke et al., 2010; Looby et al., 2014; Verdi et al., 2016; Holt and Looby, 2017; Monnet et al., 2021). Several studies have also found statistically significant relationships between academic self-efficacy and PCE usage (Looby et al., 2014), however, several studies have not found such a relationship (Verdi et al., 2016; Holt and Looby, 2017). Low academic self-efficacy is less predictive of PCE usage than low marks are. Verdi et al. (2016) capture the picture succinctly: “while actual academic failure may be a risk factor for non-medical stimulant use among university students, subjective academic stress does not appear to be a risk factor, at least among graduate students” (p. 750).

The COVID-19 lockdown measures’ impact on academic institutions forced students to adapt to home-based learning and in many cases reduced academic support. To date, no study has been published regarding the impact of COVID-19 social restrictions on specifically the use of PCEs. However, several studies have investigated changes in trends in substance use before and during COVID-19 lockdowns. There has been an increase in interest in drugs on social media, as captured by Arillotta et al. (2021), who observed a 9.2% increase in membership to drugs-related subreddits from December 2019 to May 2020. However, evidence from Italy (Gili et al., 2021) and Australia (Price et al., 2021) indicate a drop in recreational drug use due to accessibility issues and decreased opportunities for socialization. In contrast, several studies (Avena et al., 2021; Gili et al., 2021; Davies et al., 2022) found an increase in the use of alcohol and benzodiazepines, as well as an increase in cannabis use in the Netherlands (van Laar et al., 2020). Despite this, however, there has also been an increase in deaths from drug overdose, linked to the side effects of public health measures to combat the spread of the virus creating and/or exacerbating existing mental health issues, including social inequalities and job uncertainty, in combination with an increase in the toxicity of drugs, particularly in the US (Macmadu et al., 2021; Nguyen and Buxton, 2021; Friedman and Hansen, 2022).

Several studies have also investigated alcohol and drug use among students and the broader population during the lockdown periods (Lechner et al., 2020; Vanderbruggen et al., 2020; Acuti Martellucci et al., 2021; Dogan-Sander et al., 2021; Jodczyk et al., 2022; Merwid-Lad et al., 2023). Almost all of these studies investigate substance use in relation to the impact of the lockdown measures on mental health. At the time of writing this report only one study was found regarding the use of PCEs during the pandemic period, focusing on Polish medical students (Merwid-Lad et al., 2023). The findings of this study indicate that 53% of students use cognitive enhancers - mostly soft enhancers, and while 31% of students indicated a rise in their use of PCEs specifically due to the impact of the pandemic, 68% reported that the pandemic had no impact on their use of cognitive enhancers.

More broadly, an increase has been observed in the use of substances, particularly alcohol and tobacco, correlated with an increase in mental health symptoms. Acuti Martellucci et al. (2021) document a general increase in the prevalence of alcohol consumption in their systematic review, including an increase of over 25% in the UK. Lechner et al. (2020) found that American students who experienced more symptoms of depression and anxiety reported greater consumption of alcohol compared to those who perceived greater social support. Data from university students in Germany (Dogan-Sander et al., 2021) shows a significant increase in depressive symptoms and a correlated increase in the use of drugs and alcohol. Among Polish university students, Jodczyk et al. (2022) report that 72% of students reported a negative mental health impact of the pandemic, and 13% reported an increase in their use of psychoactive substances, most notably tobacco and alcohol. However, this was not correlated with the increased use of cannabis products and hard drugs. An online study in Belgium (Vanderbruggen et al., 2020) also found an increase in the use of alcohol and cigarettes, but no correlation with the consumption of cannabis.

In this cross-sectional study, an online survey investigated PCE use during the first year of social restrictions and during the year directly prior. We also investigated stacking, subjective PCE effects, and several predictive factors of use. The aim was to assess potential differences in PCE usage and subjective perceptions between these two periods of time and to shed light on whether and to what extent five-factor personality traits, academic self-efficacy, and certain demographic factors could predict an increase or decrease in this use among UK students and academic staff in a higher education context. We hypothesized that there would be a significant increase in the number of people using all types of drugs during pandemic social restrictions compared to the previous year (H1). We also predicted a greater prevalence of stacking among individuals during social restrictions compared to the previous year (H2). Additionally, we expected to observe an increase in the number of individuals reporting cognitive-enhancing effects associated with drug use during the pandemic social restrictions period (H3). And, finally, we anticipated that personality traits and academic self-efficacy, along with the demographic variables of age, gender identity, and student/staff academic status, would significantly predict the use of PCE for each of the substance types during social restrictions (H4).

## 2 Method

### 2.1 Participants and design

An opportunistic sample of 991 UK university students and academic staff completed an online cross-sectional survey from July 2021 to January 2022 (see Table 1 for sample characteristics). Initially, psychology students from a university in South West England were approached using an online course credit system, and staff were contacted through the institutional mailing list and asked to circulate to colleagues through word-of-mouth. Online advertisements across social media and Reddit sub-forums were later used to expand the sample to other students and academic staff in the UK. Respondents were eligible to take part if they were 18 and above and had been a student or staff member in a UK university during the 2019/2020 and 2020/2021 academic years. Due to missing or incomplete substance use data, a final sample of 736 respondents was brought forward for analysis. Several predictive factors were under investigation, including the Five-Factor Model (FFM) of Personality (McCrae and Costa, 1987), academic self-efficacy, gender identity, age, and student/staff academic status. Outcome variables were user status across the ten PCE types (user or nonuser), whether respondents had stacked any substances from the inventory together, and subjective enhancing effects. The study was given full ethical approval by the University of Exeter Research Ethics Committee on 4th June 2021.

**Table 1:** Demographic characteristics of study participants.

Gender (*n* = 732)	*n* (*%*)	Age (SD)
Female	474 (*64.4%*)	21.8 (4.7)
Male	208 (*28.2%*)	23.1 (6.1)
Non-binary	18 (*2.4%*)	21.8 (3.5)
Prefer not to say	6 (*0.8%*)	21.6 (4.0)
Student/staff status (*n* = 732)		
Student	647 (*91.3%*)	21.5 (4.2)
Staff	56 (*8.7%*)	29.3 (22.1)

This table provides an overview of the demographic characteristics of the study participants (*n* = 732), including gender distribution, age (mean and standard deviation), and student/staff status.

### 2.2 Materials and procedure

The survey was built and hosted using Qualtrics software (Seattle, Washington/Provo, Utah) and participants gained access through a web link where they first received participant information and provided consent by selecting a checkbox. The different scales from the survey were set up to appear in random order through the Qualtrics block randomiser, to reduce order effects. Questions on PCE were developed using the 2017 Global Drug Survey module on cognitive enhancement (as reported Maier et al., 2018) as the basis for which substance types to investigate and for the definition of cognitive enhancement. PCEs were defined at the beginning of the survey as:

*‘These are any substances used explicitly to increase your cognitive performance or mood at work or while studying without medical instruction from a professional. Please also indicate if you have used sedatives to improve sleep or relaxation if the overall purpose of use was to increase your cognitive performance the next day.’*

#### 2.2.1 Bespoke PCE scale

Ten drug types were examined [modafinil, prescription stimulants, beta-blockers, alcohol, benzodiazepines, cannabis, illegal stimulants, nutraceuticals, microdose LSD (<20 μg), and microdose psilocybin (0.15–0.20 g)] alongside examples of substances belonging to these groups [e.g., prescription stimulants (methylphenidate, dextroamphetamine etc.)]. Respondents were then asked to indicate (yes/no) whether they had used these for PCE during COVID-19 social restrictions (March 2020 – February 2021) and/or during the year prior (March 2019 – February 2020).

Participants were then asked to rate where applicable the perceived effects of these substances on cognitive performance and then mood on a 3-point scale (decreased/remained the same/increased) during social restrictions compared to the year before. To assess stacking, participants were asked to mark which drugs from the inventory they had used in the same period of time (either immediately or within the same day) starting with illegal stimulants [‘During the same period as illegal stimulants (e.g., amphetamine, cocaine etc.) I have also used…’] and then moving through the list of drugs. This produces an indication of the degree to which any two substances from the inventory are used together.

#### 2.2.2 Five-factor model of personality

Respondents also completed the 50-item Five-Factor Model (FFM) of Personality (McCrae and Costa, 1987) which assesses personality across five dimensions, including openness (e.g., “I am full of ideas”), conscientiousness (e.g., “I am always prepared”), extraversion (e.g., “I start conversations”), agreeableness (e.g., “I sympathize with others’ feelings”) and neuroticism (e.g., “I get irritated easily”). Items are scored on a 5-point Likert scale ranging from ‘strongly disagree’ to ‘strongly agree’, and there are 10 items for each dimension. Summed composite scores are produced for each subscale with higher values showing greater alignment to each factor label. The FFM has been used widely and is highly regarded in personality science and related disciplines as a reliable measure (Widiger, 2017). In this study, Cronbach’s alpha reliability coefficients for subscales on openness (*α* = 0.80), conscientiousness (*α* = 0.81), extraversion (*α* = 0.85), agreeableness (*α* = 0.86), and neuroticism (*α* = 0.83) show strong internal consistency.

#### 2.2.3 Academic self-efficacy measure

Participants were also asked about beliefs in personal academic ability and completed the academic self-efficacy measure (ASEM) (McIlroy et al., 2015). This is a short 10-item survey (e.g., “I am confident I can achieve good exam results if I put my mind to it”), with items scored on a 7-point Likert scale ranging from ‘very strongly disagree’ to ‘very strongly agree’. A single summed composite score is produced and a higher value suggests greater levels of perceived academic competence. Here, Cronbach’s alpha shows good (*α* = 0.73) internal consistency.

After completing all scales, respondents were given the option to provide a personal email address via a web link to be entered into a £50 retail voucher prize draw. Participants were then provided with a written debrief and instructed that they could close the tab.

### 2.3 Data analysis

Sample demographics were indicated with number (N), mean (M), standard deviation (SD), and percentage (%) respectively. Self-reported substance use is indicated with N and %, and Pearson’s chi-square cross-tabulations assessed significant differences in user rates during social restrictions compared with the year prior. Stacking for the two periods was indicated with N and % and presented in a heat map. Perceived effects on cognitive enhancement were shown in stacked bar charts and each drug had two adjacent columns showing data from the two time periods. We conducted a series of stepwise (forward procedure) binary logistic regression models to examine predictors of use for the 10 substance types coded as dichotomous variables (user or nonuser) during social restrictions. Potential predictors were: age, gender identity (female, male), openness, conscientiousness, extraversion, agreeableness, neuroticism, academic self-efficacy, and student/staff status. Beta (β) values, odds ratios (β exp), log-likelihood, Nagelkerke *R*^2^ and Cox and Snell pseudo *R*^2^ across model stages, and overall model significance were presented in several tables (see Table 4).

## 3 Results

### 3.1 PCE prevalence

Table 2 shows the percentage of the sample who used PCE during the first year of pandemic social restrictions compared with the year previous. For the year before social restrictions, the most popular substances in descending order were alcohol (52.5%) and cannabis (26.4%), followed by illegal stimulants (18.2%), benzodiazepines (17.1%), beta-blockers (14.8%) and nutraceuticals (14.8%), prescription stimulants (13.4%), microdose psilocybin (13.3%), microdose LSD (12.0%), and finally modafinil (11.2%). There were several changes to this order during restrictions, where in descending order the next most popular after alcohol (55.7%) and cannabis (30.7%) were benzodiazepines (19.4%), nutraceuticals (19.2%), illegal stimulants (19.0%), beta-blockers (17.2%), modafinil (16.0%), microdose psilocybin (15.7%), prescription stimulants (15.2%) and finally microdose LSD (14.5%).

**Table 2:** Changes in self-reported substance use for PCE before and during COVID-19 social restrictions.

	Year before social restrictions *n* (*%*)	During social restrictions *n* (*%*)	Change *n* (*%*)	*X* ^2^	*P*
Prescription stimulants	99 (*13.4%*)	112 (*15.2%*)	+13 (*13.1%*)	166.75	*p* < 0.001
Modafinil	83 (*11.2%*)	118 (*16.0%*)	+35 (*42.1%*)	167.03	*p* < 0.001
Beta blockers	109 (*14.8%*)	127 (*17.2%*)	+18 (*16.5%*)	175.17	*p* < 0.001
Benzodiazepines	126 (*17.1%*)	143 (*19.4%*)	+17 (*13.4%*)	138.12	*p* < 0.001
Alcohol	387 (*52.5%*)	410 (*55.7%*)	+23 (*5.9%*)	325.57	*p* < 0.001
Cannabis	195 (*26.4%*)	226 (*30.7%*)	+31 (*15.8%*)	210.48	*p* < 0.001
Illegal stimulants	134 (*18.2%*)	140 (*19.0%*)	+6 (*3.0%*)	169.60	*p* < 0.001
Nutraceuticals	109 (*14.8%*)	142 (*19.2%*)	+33 (*30.2%*)	224.47	*p* < 0.001
Microdose LSD	89 (*12.0%*)	107 (*14.5%*)	+18 (*22.2%*)	119.11	*p* < 0.001
Microdose Psilocybin	98 (*13.3%*)	116 (*15.7%*)	+18 (*18.3%*)	208.65	*p* < 0.001

This table presents self-reported substance use for cognitive enhancement before and during COVID-19 social restrictions (*n* = 736). The data includes the number and percentage of participants reporting the use of various substances, such as prescription stimulants, modafinil, beta blockers, benzodiazepines, alcohol, cannabis, illegal stimulants, nutraceuticals, microdose LSD, and microdose Psilocybin.

Data was also collected comparing the use of the 10 drug types between these periods, and chi-square cross-tabulations revealed highly significant effects across all substances, with respondents more likely to use each drug during social restrictions compared with the previous year. Although a similar number of respondents reported using at least one substance type for PCE during social restrictions (69.1%) and the year prior (68.3%), the number of individuals using specific substances increased significantly, with some drugs showing large increases. Overall, modafinil use increased the most in the sample (+42.1%), followed by nutraceuticals (+30.2%) and microdose LSD (+22.2%). Microdose psilocybin (+18.3%), beta-blockers (+16.5%), cannabis (+15.8%), benzodiazepines (+14.4%), and prescription stimulants (+13.1%) increased at similar rates, while alcohol (+5.9%) and illegal stimulants (+3.0%) showed only marginal increases.

### 3.2 Stacking

Table 3 shows a heatmap of 45 substance pairs used together for PCE the year before social restrictions compared with the period during social restrictions. Stacking for most substance pairs was between 10 to 15% with only a few exceptions. Alcohol was stacked with other drugs the most, including cannabis (23.0%), prescription stimulants (21.7%), illegal stimulants (19.2%), beta-blockers (17.9%), benzodiazepines (17.5%) and modafinil (16.4%). Illegal stimulants and cannabis (15.5%) were also used more frequently together. Modafinil was stacked the least, particularly with prescription stimulants (7.5%) and benzodiazepines (10.3%). MD Psilocybin and MD LSD were also stacked less frequently (9.9%).

**Table 3:** Heat map of substances stacked within the same day for PCE before and during social restrictions.

Heat map of substances stacked within the same day for PCE before (left section; red) and during (right section; blue) social restrictions (*n* = 736)										
	**1.**	**2.**	**3.**	**4.**	**5.**	**6.**	**7.**	**8.**	**9.**	**10.**
1. Prescription stimulants	–	146 (19.8*%*)	84 (*11.4%*)	11 (15.1%)	175 (23.8%)	115 (15.6%)	92 (12.5%)	117 (15.9%)	89 (12.1%)	85 (11.5%)
2. Modafinil	55 (7.5*%*)	–	70 (9.5%)	108 (14.7%)	120 (16.3%)	105 (14.3%)	81 (11.0%)	109 (14.8%)	86 (11.7%)	81 (11.0%)
3. Beta-blockers	94 (*12.8%*)	92 (*12.5%*)	–	116 (15.8%)	118 (16.0%)	94 (12.8%)	79 (10.7%)	100 (13.6%)	83 (11.3%)	80 (10.9%)
4. Benzodiazepines	97 (*13.2%*)	76 (*10.3%*)	88 (*12.0%*)	–	126 (17.1%)	104 (14.1%)	85 (11.5%)	110 (14.9%)	68 (9.2%)	74 (10.1%)
5. Alcohol	160 (*21.7%*)	121 (*16.4%*)	132 (*17.9%*)	129 (*17.5%*)	–	192 (26.1%)	118 (16.0%)	109 (14.8%)	77 (10.5%)	74 (10.1%)
6. Cannabis	106 (*14.4%*)	94 (*12.8%*)	81 (*11.0%*)	90 (*12.2%*)	169 (*23.0%*)	–	113 (15.4%)	93 (12.6%)	82 (11.1%)	78 (10.6%)
7. Illegal stimulants	111 (*15.1%*)	89 (*12.1%*)	91 (*12.4%*)	92 (*12.5%*)	141 (*19.2%*)	114 (*15.5%*)	–	96 (13.0%)	78 (10.6%)	66 (9.0%)
8. Nutraceuticals	87 (*11.8%*)	99 (*13.5%*)	82 (*11.1%*)	79 (*10.7%*)	102 (*13.9%*)	94 (*12.8%*)	92 (*12.5%*)	–	80 (10.9%)	71 (9.6%)
9. Microdose LSD	94 (*12.8%*)	96 (*13.0%*)	92 (*12.5%*)	97 (*13.2%*)	88 (*12.0%*)	96 (*13.0%*)	85 (*11.5%*)	84 (*11.4%*)	–	76 (10.3%)
10. Microdose Psilocybin	86 (*11.7%*)	86 (*11.7%*)	79 (*10.7%*)	81 (*11.0%*)	82 (*11.1%*)	86 (*11.7%*)	90 (*12.2%*)	85 (*11.5%*)	73 (9.9*%*)	–

Substances stacked before social restrictions					Substances stacked during social restrictions				
0-4%	5-10%	11-15%	16-20%	21%+	0-4%	5-10%	11-15%	16-20%	21%+

The heat map visualizes the concurrent use of substances for cognitive enhancement (PCE) before and during social restrictions. The table represents a matrix where each row and column correspond to a specific substance, and the cells indicate the percentage of participants reporting simultaneous use of substances within the same day. Percentages are based on the total number of participants (*n* = 736) and provide insights into the patterns of substance stacking before and during the implementation of social restrictions. The left section of the heat map shows the concurrent use of substances for PCE before social restrictions in red. The right section of the heat map illustrates the use of substances during social restriction in blue. As detailed in the table description, the percentages refer to the percentage of the total sample that stacked each substance pair. For instance, for the Modafinil-Prescription stimulant pair, 7.5% of the sample reported stacking before social restrictions.

For the period during social restrictions, stacking with most substances was between 10 to 15%, although there were several notable deviations from the previous year. There was a clear increase in the stacking of 13 pairs, while stacking of another 13 pairs remained roughly at the same level between the two periods (less than 1% difference) and stacking of 18 substance pairs showed a clear decrease. Alcohol was still stacked the most, including with cannabis (26.1%), prescription stimulants (23.8%), benzodiazepines (17.1%), modafinil (16.3%), illegal stimulants (16.0%) and beta-blockers (16.0%). However, there was a notable rise in prescription stimulant stacking, especially with modafinil (19.8%), followed by nutraceuticals (15.9%) and cannabis (15.6%). Modafinil and benzodiazepine stacking also notably increased (14.7%). MD Psilocybin stacking fell from the previous year, particularly with MD LSD (10.3%), benzodiazepines (10.1%), alcohol (10.1%), nutraceuticals (9.6%), and illegal stimulants (9.0%). Stacking of MD LSD and benzodiazepines also fell (9.2%), as did modafinil and beta-blockers (9.5%).

### 3.3 Perceived effects on cognition

**Figure 1:**
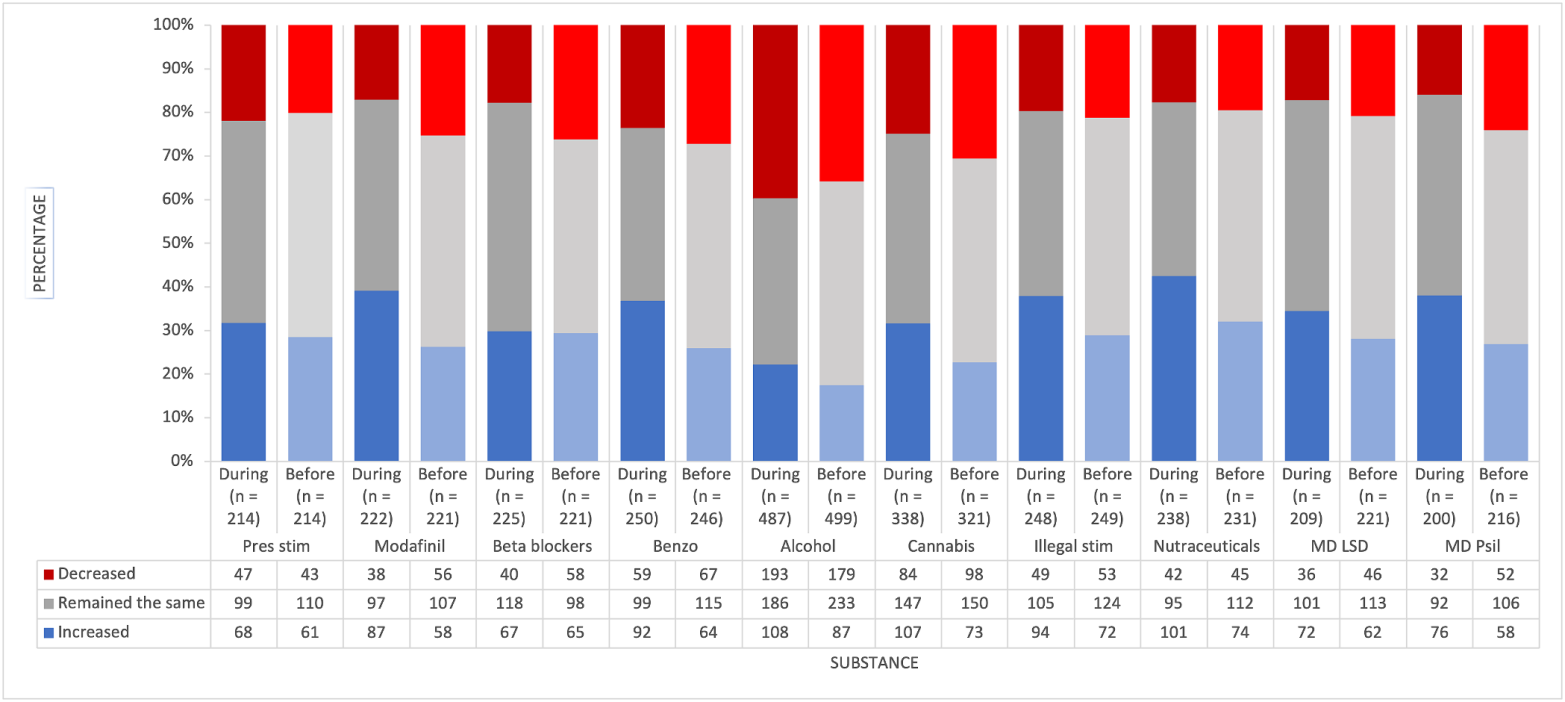
Self-reported perceived PCE for different substance types during and before social restrictions. The figure displays the self-reported perceived performance-enhancing effects (PCE) of various substances before and during social restrictions. The substances include prescription stimulants, modafinil, beta blockers, benzodiazepines, alcohol, cannabis, illegal stimulants, nutraceuticals, microdose LSD, and microdose psilocybin. Each bar represents the percentage of participants who reported an increase (blue), decrease (red), or no change (grey) in perceived PCE. The number of respondents for each substance is indicated below the respective bars.

Figure 1 displays categorical data for perceived effects of substances used for PCE during and before social restrictions. Overall, a greater proportion of respondents reported increased PCE across all drug types during social restrictions than the previous year. Modafinil showed the greatest perceived cognitive effect increase (+50.0%), followed closely by cannabis (+46.6%) and benzodiazepines (+43.6%). Fewer people also reported decreased cognitive effects for these drugs than the year previous, revealing positive overall user perceptions. Nutraceuticals (+36.5%), MD psilocybin (+31.0%) and illegal stimulants (+30.0%) also showed similar increases, although reports of decreased performance fell only slightly compared with the previous year for nutraceuticals (−6.6%) and illegal stimulants (−7.5%). Alcohol had a mixed profile, as increased cognitive effects were up (+24.1%) from the previous year, but so too were decreased effects (+9.8%). MD LSD (+16.1%) and prescription stimulants (+11.5%) both showed moderate cognitive effect increases, although reports of decreased performance showed a nominal increase with prescription stimulants (+9.3%). Beta-blockers had the smallest cognitive effect increase (+3.1%), but notably, fewer people reported decreased performance (−31.0%) from the previous year.

### 3.4 Predictors of use

Table 4 presents the results of 10 binary stepwise logistic regression analyses predicting the use of various drug types during social restrictions. The models included FFM personality traits, academic self-efficacy, gender identity, age, and student/staff status as potential factors in each analysis. All regression analyses were highly significant at the final step (*p*s < 0.001), and eight of the predictive models demonstrated a good fit with the data, while two exhibited a moderate fit. In descending order, the substances with the highest Nagelkerke R2 (%) explained variance and demonstrating a good fit, were modafinil (38.2%), nutraceuticals (34.1%), microdose psilocybin (33.8%), prescription stimulants (33.8%), beta blockers (27.7%), microdose LSD (25.2%), illegal stimulants (20.6%), and benzodiazepines (19.4%). Both cannabis (12.5%) and alcohol (4.7%) showed comparatively smaller, moderate fits.

**Table 4:** Stepwise binary logistic regression assessing predictors of the 10 drug types during COVID-19 social restrictions (*N* = 625).

								Final model			
Outcome variable	Step	Regressor	*R*^2^ C&S	*R*^2^ NK	Log likelihood	*X* ^2^	df	β	β [exp]	Wald	*p*
Modafinil											
	1	Agreeableness	0.188	0.322	416.33	129.93	1	−0.192	0.826	64.52	<0.001
	2	Gender	0.204	0.350	403.84	142.42	2	−0.738	0.478	8.17	0.004
	3	Conscientiousness	0.216	0.371	349.03	152.23	3	−0.078	0.925	10.54	0.001
	4	Neuroticism	0.223	0.382	388.81	157.45	4	0.050	1.052	5.17	0.023
Prescription stimulant											
	1	Agreeableness	0.140	0.230	487.66	93.94	1	−0.144	0.866	57.77	<0.001
	2	Age	0.156	0.257	475.69	105.90	2	0.066	1.069	12.01	<0.001
	3	Conscientiousness	0.168	0.277	466.80	114.79	3	−0.059	0.943	8.67	0.003
Beta blocker											
	1	Agreeableness	0.140	0.230	487.66	93.94	1	−0.144	0.866	57.77	<0.001
	2	Age	0.156	0.257	475.69	105.90	2	0.066	1.069	12.01	<0.001
	3	Conscientiousness	0.168	0.277	466.80	114.79	3	−0.059	0.943	8.67	0.003
Benzodiazepine											
	1	Agreeableness	0.103	0.163	557.58	67.92	1	−0.107	0.898	41.59	<0.001
	2	Conscientiousness	0.115	0.181	549.40	76.10	2	−0.054	0.948	8.90	0.003
	3	Extraversion	0.122	0.194	543.92	81.59	3	−0.038	0.962	5.41	0.020
Alcohol											
	1	Extraversion	0.011	0.015	854.68	6.91	1	0.039	1.039	11.44	<0.001
	2	Neuroticism	0.022	0.030	847.51	14.07	2	−0.040	0.961	10.57	0.001
	3	Gender	0.035	0.047	839.23	22.36	3	−0.525	0.592	8.13	0.004
Cannabis											
	1	Conscientiousness	0.046	0.065	726.61	29.19	1	−0.061	0.912	14.79	<0.001
	2	Extraversion	0.062	0.089	715.72	40.07	2	0.048	1.049	11.69	<0.001
	3	Agreeableness	0.072	0.103	709.12	46.68	3	−0.050	0.951	10.90	<0.001
	4	Openness	0.082	0.117	702.25	53.55	4	0.054	1.056	9.09	0.003
	5	Academic self-efficacy	0.088	0.125	698.32	57.49	5	−0.039	0.962	3.93	0.047
Illegal stimulant											
	1	Agreeableness	0.071	0.117	538.51	45.96	1	−0.096	0.908	28.61	<0.001
	2	Conscientiousness	0.105	0.173	515.14	69.53	2	−0.084	0.920	19.04	<0.001
	3	Extraversion	0.116	0.192	508.36	77.32	3	0.053	1.054	8.22	0.004
	4	Gender	0.125	0.206	501.25	83.42	4	−0.575	0.563	6.15	0.013
Nutraceutical											
	1	Agreeableness	0.156	0.248	514.04	105.88	1	−0.151	0.860	61.05	<0.001
	2	Gender	0.193	0.307	485.86	134.07	2	−1.176	0.308	25.59	<0.001
	3	Age	0.215	0.341	468.89	151.03	3	0.078	1.081	15.98	<0.001
MD LSD											
	1	Agreeableness	0.126	0.227	423.51	84.50	1	−0.154	0.858	51.26	<0.001
	2	Gender	0.135	0.242	417.61	90.40	2	−0.554	0.574	4.81	0.028
	3	Conscientiousness	0.140	0.252	413.74	94.27	3	−0.043	0.958	3.82	0.051
MD Psilocybin											
	1	Agreeableness	0.180	0.306	428.99	123.90	1	−0.186	0.830	71.46	<0.001
	2	Conscientiousness	0.192	0.326	419.95	132.94	2	−0.065	0.937	8.49	0.004
	3	Age	0.198	0.338	414.86	138.03	3	0.047	1.048	5.23	0.022

C&S, Cox & Snell; NK, Nagelkerke; MD LSD, Microdose LSD; MD Psilocybin, Microdose Psilocybin.

Regressor-level statistics revealed several consistent predictive factors that explained a significant amount of variance for more than half of the predictive models. Agreeableness was the most consistent predictor, as a one-unit decrease in score was associated with increased odds of using modafinil (17.4%), microdose psilocybin (17.0%), prescription stimulants (16.2%), microdose LSD (14.2%), nutraceuticals (14.0%), beta blockers (13.4%), benzodiazepines (10.4%), illegal stimulants (9.2%), and cannabis (4.9%). Gender identity was also a consistent predictor, with identifying as male associated with increased user odds for nutraceuticals (69.2%), prescription stimulants (60.3%), modafinil (52%), illegal stimulants (43.7%), microdose LSD (42.6%), and alcohol (41.8%). Another consistent predictor was conscientiousness, as a one-unit decrease in score was linked to increased odds of using cannabis (8.8%), illegal stimulants (8.0%), modafinil (7.5%), MD psilocybin (6.3%), beta-blockers (5.7%), and benzodiazepines (5.2%).

Several predictor variables were less consistent and explained a significant amount of variance in fewer than half of the various models. A one-unit increase in extraversion score was associated with increased odds for illegal stimulants (5.4%), cannabis (4.9%), and alcohol (3.9%), but a one-unit decrease in score was linked to increasing odds for benzodiazepine use (3.8%). With age, a one-unit increase was linked to increasing user odds for nutraceuticals (8.1%), beta-blockers (6.9%), and microdose psilocybin (4.8%). For neuroticism, a one-unit increase in score increased user odds for modafinil (5.2%), but a one-unit decrease was linked to increased odds for alcohol (3.9%). Furthermore, a one-unit increase in openness (5.6%) and academic self-efficacy (3.8%) scores were associated with increasing cannabis use only, and student/staff status was not a significant predictor in any of the models.

## 4 Discussion

We have reported findings from a PCE use survey of students and staff in UK universities and this is the first survey of its kind to investigate the use of these strategies during the COVID-19 pandemic. We defined PCE as the use of any substance with the explicit intention to increase cognitive performance for work or study without prior medical instruction. Our inventory included 10 drug types (modafinil, prescription stimulants, beta-blockers, alcohol, benzodiazepines, cannabis, illegal stimulants, nutraceuticals, microdose LSD, and microdose psilocybin) that had been reported on previously in the Global Drug Survey. The research had four objectives, assessing: (a) prevalence, (b) stacking, (c) perceived effects, and (d) predictive factors. Our findings revealed several key insights into PCE use during the first year of pandemic social restrictions and some noteworthy differences with the year directly prior.

### 4.1 PCE usage

We expected to see a significant increase in the number of people using all drug types during social restrictions compared with the previous year (H1), and this prediction was supported. Two-thirds of our sample reported some kind of PCE usage during both time periods, but slightly more respondents reported use during social restrictions. All drug types showed increases in the number of respondents reporting use during social restrictions, and the largest increases were observed with modafinil, nutraceuticals, and microdose LSD. Of note was a considerable increase in modafinil use (+42.1%), which aligns with findings from previous research demonstrating an increase in modafinil consumption at UK universities over the last two decades (Sharif et al., 2021). Nevertheless, pandemic restrictions might be linked to this sudden increase as more people potentially turned to modafinil to cope with changing study and work demands. The availability of modafinil via online pharmacies could be another factor behind the observed increase. A review published during the pandemic period concluded that modafinil was widely available for purchase across 203 websites that deliver within the UK, and that pricing per tablet (£0.38–5.31) was cheap to encourage purchase (Hockenhull et al., 2020). We also observed a significant increase in nutraceutical (+30.2%) use. Their wide availability and ease of purchase could explain their increased popularity during this time when there were changes in access to illegal drug markets brought about by national lockdowns (Hawdon et al., 2022) as people adapted to changing market conditions and sought replacement strategies. Microdose LSD also exhibited a significant increase (+22.2%) in our sample, which is consistent with research demonstrating recent growth in the popularity of microdosing psychedelics (Anderson et al., 2019). This could be attributed in part to a wider trend some have termed the “Psychedelic Renaissance,” which has created renewed interest in the therapeutic potential of psychedelics and has yet to show signs of slowing (Sessa, 2012, 2018).

### 4.2 Stacking

We also hypothesized that the prevalence of stacking would increase during pandemic social restrictions compared to the previous year (H2), and this was partially supported. Overall stacking of the different substances in our inventory was at the 10 to 15% range both before and during the restrictions in most cases; however, differences were observed in which substance pairs were stacked in the two periods. Of the 45 pairs assessed in our study, stacking increased in 13 pairs, remained generally at the same level for another 13 pairs, and decreased in 18 pairs. Stacking of illegal stimulants, MD LSD, and MD psilocybin with all other substances in our inventory either remained the same or decreased during social restrictions, which could be indicative of accessibility issues mentioned above (Hawdon et al., 2022). Otherwise, stacking of prescription stimulants, benzodiazepines, and cannabis increased with 5 other substances each, and stacking of nutraceuticals and modafinil increased with 4 other substances each. Stacking profiles of alcohol pairings and beta-blocker pairings are more mixed as stacking of alcohol increased with 2 other substances (one of which is cannabis, constituting the most prevalent pair of stacked substances in both time periods), remained roughly the same with 3, and decreased with 4, while stacking of beta blockers increased with 3 substances and fell with 5 other substances.

Despite this, alcohol remained the substance most commonly combined with others across both periods. This observation is unsurprising, given the prevalence of alcohol in our sample and other meta-analysis data indicating a general increase in alcohol polydrug use during the early pandemic time period (Roberts et al., 2021). Furthermore, MD psilocybin was stacked the least overall, followed closely by MD LSD, and the MD LSD-benzodiazepine pair showed the biggest fall in stacking overall (from 13.2 to 9.2%). In one sense, this is surprising, considering the recent surge in popularity of psychedelics (Sessa, 2012; Sessa, 2018). However, it could equally be attributed to these substances not being well-tolerated in combination with other drugs. This argument is strengthened by the fact that when stacking is not considered, both MD LSD and MD psilocybin showed the third (+22.2%) and fourth (+18.3%) highest increase, respectively. The most drastic increase in stacking prevalence was observed in the modafinil-prescription stimulant pair, increasing from 7.5 to 19.8%. This appears to be linked to increasing modafinil user trends detailed above (Sharif et al., 2021) which might have been accelerated by pandemic restrictions, but also pharmacokinetics which show that modafinil is tolerated well with other stimulants (Hashemian and Farhadi, 2020).

This is the first study to perform a detailed investigation into PCE stacking and also to draw comparisons between the first year of pandemic social restrictions and the previous year. Hence, it is difficult to determine whether the observed stacking rates are in any way typical or if changes between the time periods reflect a larger trend. Some evidence does indicate an increase in stacking among adolescents in the previous decade (Zuckermann et al., 2019) but our findings show that stacking during the lockdown period was similar to the preceding year.

### 4.3 Perceived enhancing effects

We predicted that the number of people reporting enhanced cognitive effects associated with drug use would increase during social restrictions (H3), and this was supported. Regarding subjective PCE, across all drug types assessed in this study, a higher proportion of participants reported increased performance and a smaller proportion reported decreased performance, revealing a positive perceived effect profile for each drug type, except alcohol, which had a mixed profile. A possible explanation for these findings could be that respondents were able to reflect more on the experience of using various PCE during social restrictions and this generally increased positive perceptions. Modafinil, benzodiazepines, and cannabis had the highest increase in subjective PCE (>40%), indicative of several plausible explanations. One interpretation is that these substances are simply best at reducing pandemic-related cognitive pressures. Another perspective is that they have better subjective cognitive perceptions when used in solitary environments when students and staff could better focus on study and work, as was likely to be the case during social restrictions. Even alcohol, a substance well-known for impairing cognitive functioning (Field et al., 2008), showed an increase in reported PCE, although reports of decreased performance also increased. A possible explanation for this is that respondents reporting increased PCE used only small doses, and in limited quantities, alcohol is known to function as a central nervous system stimulant and can increase feelings of psychological resilience (Hendler et al., 2011). Our study did not capture dosage information for any substances used. This limits our ability to determine if the perceived effects of PCE were linked to the quantity consumed. It is possible that respondents increased their PCE dosage during social restrictions as a coping mechanism, as previously posited. However, without data on dosage, we cannot make this claim. Additionally, the questions on perceived PCE only assessed use of single substances, not combinations. Some drugs, when combined, can potentially show amplified effects. Nevertheless, the observed increase in perceived PCE effects during social restrictions did not coincide with a significant rise in reported stacking. This suggests that stacking alone cannot be the primary explanation for these observations.

### 4.4 Predictors of use

Our final hypothesis was that personality traits, academic self-efficacy, along with the demographic variables of age, gender identity, and student/staff academic status would predict the use of PCE for each of the substance types during social restrictions (H4), and this, too, was partially supported. The clearest predictors that emerged out of our analysis are agreeableness, and gender, followed by conscientiousness. Agreeableness scores in particular proved to be a strong predictor, as the analysis returned highly significant results for 9 out of the 10 drug types assessed here (except alcohol), and as the strongest predictor in 8 of them (except cannabis). In descending order, decreased agreeableness was associated with rising odds of using modafinil, microdose psilocybin, prescription stimulants, microdose LSD, nutraceuticals, beta blockers, benzodiazepines, illegal stimulants, and finally cannabis. This aligns with recent data (Sharif et al., 2021) showing that lower levels of agreeableness predict CE usage, more so than low conscientiousness and high neuroticism. Furthermore, a possible explanation in our study is that respondents scoring lower in agreeableness were more likely to engage with CE use during the lockdown as a form of rebellion and novelty seeking to cope with pandemic pressures.

The second key predictor was gender, which was a significant factor concerning the use of 6 of the 10 types of drugs assessed here. In descending order, being male increased the odds of using nutraceuticals, prescription stimulants, modafinil, illegal stimulants, microdose LSD, and alcohol. This is an unsurprising finding, as gender differences in drug use are already well established in that men are more likely to engage in almost all types of drug use compared to women (Fattore et al., 2020), and pandemic restrictions appear not to have altered this fact.

In line with the extant literature (Sattler and Schunck, 2016; Sharif et al., 2021), conscientiousness was another strong predictor for the majority of substances examined. This was a highly significant predictor concerning the use of 6 of the 10 drug types assessed in this study, although the variance explained was notably smaller. In descending order, decreased conscientiousness was linked with increased odds of using cannabis, illegal stimulants, modafinil, MD psilocybin, beta-blockers, and benzodiazepines.

In contrast to this, neuroticism turned out to be a significant predictor of CE use regarding only two of the drug types assessed here - alcohol and modafinil, a clear divergence from existing literature as neuroticism has been previously linked to CE use broadly (Sattler and Schunck, 2016). Moreover, personality and demographic traits that were not consistent predictors include extraversion, which was a significant predictor of only benzodiazepine, alcohol, cannabis and illegal stimulant use; age, which was a significant predictor of MD psilocybin, nutraceutical and beta-blocker use; openness, which only predicted cannabis use; academic self-efficacy which also only predicted cannabis use; and student/staff status which was not a significant predictor for the use of any type of drug at all. It is unclear why these differences exist, although it is clear that the pandemic uniquely impacted CE more generally, hence some differences in predictive factors are unsurprisingly.

## 5 Limitations and future directions

Finally, a number of limitations need to be acknowledged within the current study. The most obvious is that the survey was self-selected and we relied on self-report measures for analysis. As a result, the true extent of PCE use in the participating universities is not fully known and respondents are not wholly representative of the general student and staff population. Furthermore, as the study was advertised on social media and Reddit, we cannot discount the possibility that respondents who were not students or staff in the UK completed the survey. Additionally, this survey was designed for people who use a range of substances for PCE, thus findings cannot be generalised outside of this population. The majority of respondents were also female (64.4%), and as highlighted above, men are more likely to use almost all drug-types compared to women (Fattore et al., 2020), hence it is possible that the female leaning sample under-represented the true extent of PCE use in the wider population. Although differences are small, the FFM has also been shown to vary between genders, with women reporting higher trait agreeableness to men (Weisberg et al., 2011; Rahmani and Lavasani, 2012). Consequently, although lower agreeableness was a consistent predictor of PCE use in our study, this finding might be inflated by the gender biassed sample and thus more relevant for women who show lower levels of agreeableness than for men. Another drawback is that we did not collect data on dosage or frequency of use. Therefore, we are limited in the inferences that can be drawn from the findings. The data cannot distinguish between people who experimented once and those who have an established pattern of drug use. This also limits the generalizability of our predictive models, as we cannot determine factors that influence high-dose and frequent users. Additionally, the survey did not capture data on recreational or medical use, so we are unable to say how PCE usage compares to these other kinds of use. Furthermore, recall bias might have affected the accuracy of self-report as participants had to recall patterns of substance use over a 2-year period. Deliberate misreporting is another consideration, and some respondents might have provided socially desirable responses. Finally, there could have been incidences in which the same respondents completed the survey more than once, although there were no duplicate response sets found in the analysis to suggest that this is the case.

The findings and subsequent limitations of this study point to a number of implications for future research in the area of PCE. Primarily, there is a pressing need for further investigations into PCE use at the university level as the significant rise we charted over the pandemic period underscores an upward trend in the use of multiple drug types. Now that social restrictions and much of the pandemic upheaval have subsided, it is crucial to establish whether rising PCE trends have persisted or if they have reverted to pre-pandemic levels. Conducting a study focused on the post-pandemic period would be a valuable contribution to addressing this question. Furthermore, the evidence from this study revealed a consistent relationship between low trait agreeableness and PCE use. Follow-up research should examine this association further, to establish if low agreeableness is a risk factor that transcends the pandemic period and if this is an important predictor for women and men. Comparative analyses across different groups might also prove useful in determining if current trends extend beyond universities to the wider population and in assessing whether use and motivations vary across different professions and industries. Cross-country analyses are also valuable (Maier et al., 2018) since nations with different cultural, legal, and healthcare systems might exhibit differences in prevalence and attitudes toward PCE. In particular, comparisons between countries that adopted different policy stances on pandemic social restrictions could prove useful. A future study focusing on the predictive factors of dosage and frequency of PCE use would also be valuable and further build on the findings from this study. Collecting data on recreational and medical use alongside PCE use would allow for valuable comparisons. This would provide insight into the specific patterns of PCE use compared to drugs used recreationally or medically. Finally, university governing bodies should take note of rising PCE trends and take proactive steps to address the issue by reviewing policies and procedures while prioritising their pastoral responsibilities to students and staff. Discussions around workloading and provisions for mental health support should be key approaches to this issue rather than restrictive and punitive measures.

## 6 Conclusion

This research investigating PCE use in students and staff at UK universities during the recent COVID-19 pandemic has made several noteworthy discoveries. One of the most significant findings to emerge is that PCE usage increased significantly across all drug-types in the first year of social restrictions compared to the previous year, and these increases were most pronounced with modafinil, nutraceuticals and microdose LSD. Moreover, perceived PCE effects were greater for all substances. Drug stacking revealed a more nuanced picture with 13 substance pairs increasing on the previous year but 18 pairs decreasing. However, there was a notable increase in the prescription stimulant-modafinil pairing. Predictive factors varied between drug-type, but low agreeableness, identifying as male, and low conscientiousness emerged as frequent predictor variables. The COVID-19 pandemic appears to have opened the door to increased PCE usage and increased perceptions that many substances are mentally beneficial. More research is required to examine whether these trends continue in the post-pandemic period, including comparative analyses across different groups and across countries to properly assess the scope of these observations. Universities should pay close attention to PCE usage and construct coherent strategies that prioritise the needs of the individual when tackling this issue.

## Data availability statement

The raw data supporting the conclusions of this article will be made available by the authors, without undue reservation.

## Ethics statement

This study involving humans was approved by the University of Exeter Research Ethics Committee. The study was conducted in accordance with the local legislation and institutional requirements. The participants provided their written informed consent to participate in this study.

## Author contributions

JT: Writing – original draft, Conceptualization, Data curation, Formal analysis, Investigation, Methodology, Writing – review & editing. OB: Writing – original draft, Investigation. JR: Writing – review & editing, Data curation, Formal analysis. JK: Writing – review & editing. TS: Project administration, Supervision, Writing – review & editing.
